# The Experience of Clinical Nurses after Korea’s Enactment of Workplace Anti-Bullying Legislation: A Phenomenological Study

**DOI:** 10.3390/ijerph18115711

**Published:** 2021-05-26

**Authors:** Hee-Sun Kim, In-Ok Sim

**Affiliations:** Red Cross College of Nursing, Chung-Ang University, 84 Heukseok-ro, Dongjak-Gu, Seoul 06974, Korea; loveof6416@naver.com

**Keywords:** experience, clinical nurse, workplace, anti-bullying legislation

## Abstract

Workplace bullying is a global issue that has emerged over the past decades and is widespread all around the world. In Korea, there is a high prevalence of bullying in nursing. In 2019, Korea enacted its workplace anti-bullying legislation. This study explores the changes experienced by nurses after the enactment of the legislation and identifies problems and improvements. Qualitative research was conducted using the phenomenological analysis method proposed by Van Kaam. Twelve nurses with experience in working before and after the enactment of the legislation were selected as study participants. They worked in various departments in five general hospitals across Korea. Purposive sampling was used to recruit participants. Data were collected using one-on-one interviews. These data were analyzed by extracting significant statements and classifying them into categories, themes, and subthemes. The analysis yielded 14 subthemes, five themes, and three categories. The three categories were “positive effect of the law”, “need for awareness of the law reform”, and “impracticalities of the law that caused chaos”. There is a difference between the theory of the law and how things happen in practice in actual nursing situations. Nurses have been educated about the new law, but better education with input from nurses themselves is needed. This study lays the groundwork for a quantitative study of the issue.

## 1. Introduction

Workplace bullying is a worldwide phenomenon with an increasing incidence over the last decades. Bullying in the workplace is recognized worldwide as a serious problem in social management, and interests and approaches are diversifying to prevent and manage it [1,2,3,4,5]. Workplace bullying is defined as the relationship between a perpetrator who continuously inflicts injury on another individual and the victim who is repeatedly abused. Although the severity of bullying varies depending on the working environment, it occurs in almost every occupational group, including nursing [4,6].

The consequences of bullying at work can lead to physical and mental problems by leaving people with weak powers unadaptable at work or in unethical and irrational situations in which they are unable to defend themselves [6]. Working in a safe and healthy environment is a basic right of workers. Nevertheless, power-driven violence and domination by bullying in the workplace have negative consequences at various levels in the work environment [7,8,9].

Although workplace bullying may be influenced by personal or environmental factors such as age and department [10,11,12,13], there is evidence that it is more affected by organizational factors such as the organizational culture and leadership of the supervisor, ethics, and tolerance [11,14]. Depending on the impact of these factors, workplace bullying can be categorized into different types, such as person-related and work-related [1].

Several factors affect the bullying of nurses. First, the closed culture of hospitals may create a suppressive atmosphere where harassment is tolerated, which ultimately results in low motivation to report cases of bullying. Second, owing to the nature of the nursing culture, workplace bullying is not properly recognized. Third, inappropriate perception of bullying in nursing, where mistreatment can occur throughout the process of becoming a nurse, hinders further actions to eradicate the problem [15].

Workplace bullying affects the quality of work, and those who experience it suffer from physical and mental distress [12,16]. Numerous studies have reported these symptoms as major factors that increase the intent to leave the job [10,17,18,19,20,21,22]. Therefore, since workplace bullying in the field of nursing causes the working environment to deteriorate [23] by fostering job dissatisfaction, intent to leave, high absence rates, low productivity, and medication administration errors, there is an urgent need to address this issue with appropriate measures [24].

Workplace bullying has drawn public attention in Korea since the country’s air rage incident (known as the “nut rage incident”) in 2014, the assault of the owners of IT companies in 2018, and nurses’ suicides in 2018 resulting from the culture of workplace hazing (known as “taeum”*,* lit. “burn-to-ashes”) [11,13,14]. According to a recent survey, 73.3% of employees have experienced various forms of bullying in the workplace [15]. Similarly, a report by the Korea Labor Institute found that 66.3% of employees have experienced harassment [8]. In the context of this study, instances of bullying of nurses are 41.3% higher than those in other occupations [17], and 65% of nurses have experienced bullying while on duty [18].

Before the enactment of the workplace anti-bullying law in Korea, judgments treated charges of workplace harassment as specific acts related to causing injury, assault, threatening behavior, defamation, using insults, and coercion [25]. However, these charges not only failed to address various types of workplace bullying, but also limited further prevention and supervision. This problem has led to the need to revise the Labor Standards Act for workplace harassment. Since 2013, various bills related to workplace harassment issues have been submitted to the National Assembly. On 18 July 2018, the government announced the Anti-Bullying Action Plan, which set out six stages and 21 items containing tasks to respond to and eradicate workplace harassment. The plan was enacted on 16 July 2019. The content of the Work Prohibition Act is: “A worker shall not inflict physical or mental pain on other workers by using the superiority of his or her position or relationship at work, or deteriorate the working environment beyond the scope suitable for the job”. The Act also states: “In addition, anyone who learns that harassment has occurred at work can report it to the employer, and if you receive a report or learn that harassment has occurred at work, you will be investigated to confirm the facts without delay. And, if necessary, to protect workers who claim to have been injured or harmed, appropriate measures, such as job changes and paid leave orders, must be taken” (Labor Standards Act system: Article 76-2, Article 3) [26]. Since then, the Ministry of Employment and Labor (MOEL) has reviewed its effectiveness and introduced several improvements, including the expansion of protected targets, the establishment of bullying reporting counters, and a review of measures to prevent them in advance [27]. In addition, iterative and diverse studies are underway to identify and rectify issues in order to continue to consider additional laws on harassment on an ongoing basis [28].

Nevertheless, especially in the nursing work environment, the physical and emotional pain of nurses due to bullying in the workplace and related negative and extreme behaviors still arise [8]. However, since thorough research on whether to solve problems in the nursing field, problems related to law, and the possibility of improvement is still insufficient, it is an urgent task to establish a theoretical foundation for in-depth improvement.

This study aims to provide preliminary data for subsequent clinical application by recognizing the changes in the bullying culture experienced by nurses in the medical environment after the establishment of a workplace bullying prevention method, and identifying potential persisting issues. To achieve this goal, this study addresses research questions such as:

(1) What have nurses recognized and experienced major changes in the field of nursing since the workplace anti-bullying legislation was enacted?
(2) What are the problems with the current workplace bullying prevention method that nurses are experiencing and what needs to be improved in the future?

## 2. Materials and Methods

### 2.1. Study Design

This qualitative research was conducted using the phenomenological analysis method proposed by Van Kaam [29]. The advantages of using Van Kaam’s [29] method include the quantitative benefits of extracting the frequency and order of meaningful statements, where the order of priority is assigned based on the frequency of the content of a specific experience. In addition, this method allows researchers to understand common aspects of the studied experiences, rather than participants’ characteristics [30].

### 2.2. Study Participants

The subjects of this study were 12 nurses working at General Hospital in Seoul, Incheon, Gyeonggi-do, and Daejeon, and were composed of nurses with experience working in hospitals before and after the workplace bullying prevention law was implemented. Participants with adequate knowledge, experience, and attitudes who could provide a comprehensive response to the research questions were recruited. Recruitment was performed using purposive sampling, which is a sampling strategy that selects participants who can provide experience-rich data related to the research question [31]. Participants were recruited among nurses with various characteristics in terms of work experience and workplace.

### 2.3. Data Collection

Data for this study were collected from 21 May 2020 to 15 June 2020. Prior to data collection, the study objectives were accurately described, and interviews were described with semistructured questions. They also explained that everything was recorded during the interview process and that the data collected was not used for nonresearch purposes, and consent was obtained for participation in the study. After obtaining prior consent, in order to create an environment where participants could feel comfortable, 1:1 interviews were conducted in individual counseling rooms and small conference rooms in the hospital, so that various in-depth experiences could be sufficiently expressed. Interviews took at least 1–2 h per person through face-to-face interviews, and additional individual interviews were repeated if necessary. After the final interview, two researchers transcribed the entire recording in writing and reaffirmed that nothing was left out of the recording.

### 2.4. Data Analysis

Data collection and analysis were performed simultaneously. In the process of data analysis, the theoretical saturation point can be determined during data collection, so the data can be analyzed with minimal sampling. To reinforce the validity of our findings, we were wary of potential research biases or biases. To ensure consistency, participants were informed in advance of the questionnaire and the direction of the study so that these actions could be perceived as a starting point for data collection. The questionnaire only contained questions about issues related to anti-bullying laws in the workplace, confirming that the researcher’s personal experience and the pilot study were not involved.

In the course of the analysis, the researchers first read the entire experience and understood the meaning. Second, after reviewing the text, representative phrases were found and highlighted in each participant’s statement. Third, researchers reorganized the highlighted annotations into a general format. Fourth, the format for describing meaningful experiences was classified as similar subjects. Fifth, a cluster of themes was derived from the categories. Finally, the collected data was summarized and explained accurately to identify the essential structure of the experience.

To ensure reliability and validity, we presented data and results from study participants to confirm systematic feedback. In addition, the study results were presented to other nurses (i.e., not study participants) working in the same environment, and the results were confirmed by them.

### 2.5. Researchers’ Qualifications

The corresponding author completed courses on qualitative study methods at graduate school and studied relevant theories through video lectures and attending special lectures at academic conferences. The present researcher has seven years’ experience in working as a nurse in the intensive care unit at a general hospital. As a nurse, the present researcher has been specifically interested in workplace bullying in nursing and the hospital system, and has established a theoretical framework to find a solution.

### 2.6. Ethical Considerations

The study obtained IRB approval (IRB: 2020-04-004) for qualitative research from the K Hospital in the Gyeonggi province. Efforts were made to ensure the confidentiality and anonymity of participants before data collection, and participants were informed that the answers would be audio-recorded once the interview started. Upon signing the informed consent document, participants were informed that they could withdraw from the study at any time. They were also informed that the collected data would be stored for three years and then discarded.

## 3. Results

### 3.1. General Characteristics of Study Participants

As a result of purposive sampling until the theoretical saturation point was reached, 12 nurses were selected as study participants. The age of the participants ranged from 26 to 54 years (average 33.2 years). With regard to marital status, 50% were single. Work experience ranged from 3 to 28 years (average 7.9 years), and seven participants had changed jobs during their employment history. Participants’ workplaces were in major urban areas, such as Seoul, Incheon, the Gyeonggi province, and Daejeon. As for hospital size, three workplaces were general hospitals with an advanced patient care system and two were general hospitals. The affiliated departments were internal medicine units (pulmonology, infectious disease, gastroenterology), surgical units (orthopedics, obstetrics, gynecology), and full-time departments (administration, nurse specialist).

### 3.2. Results Based on Van Kaam’s Analysis

A total of 90 significant statements were extracted from the data and classified into categories, each consisting of responses sharing common ideas. From the significant statements, common factors were gathered and reclassified into 14 subthemes, and five themes were created based on the same classification process. After reviewing the characteristics of the themes as a whole, the statements were classified into the final three categories: (1) “positive effects of the law”, (2) “need for awareness of the law reform”, and (3) “impracticalities of the law that caused chaos”. An overview of the findings is presented in Table 1.

#### 3.2.1. Category 1: Positive Effect of the Law

Category 1 referred to the positive experiences of participants after the enactment of the workplace anti-bullying law (Table 2). When the nurses first learned about the law, they expected that “harassment” in the workplace would no longer exist. In fact, the nurses did experience some improvements in their working environments. The biggest improvement was that they felt less discouraged and had a happier demeanor. In the past, mistakes resulted in a scolding, given with an offensive insult in a public space where everyone could hear. Now, instead, gentle feedback is given to the newly graduated nurses who make mistakes and lack professionalism. The nurses stated that it was rare to see a scolding of junior nurses who behaved irrationally. The nurses added that they hoped to see some changes that would allow them to work in a better environment and a friendly atmosphere. Additionally, other nurses expressed that they noticed some positive changes in nursing and the work atmosphere.

##### Changes in Organizational Culture in Nursing

The theme “changes in organizational culture in nursing” was divided into the following two subthemes: “changing nursing environment” and “experimental change”. When the nurses first learned about the workplace anti-bullying law, they expected they would no longer witness or experience hazing. Some evidence about the improvements in the work environment was that the nurses were becoming less discouraged and more active.

In addition, the nurses explained that it was rare to see the public insulting or shaming of inexperienced or newly graduated nurses for their mistakes, and, unlike in the past, there was no more pointless scolding.

The nurses said they would like to continue to work in a brighter work atmosphere through positive changes, and there were other statements showing that they could feel the difference before and after the enactment of the workplace anti-bullying law.

*“When I was a newly graduated nurse, I was yelled at, insulted, pinched, and even hit with the edge of the clipboard during handovers. Now the senior nurse has been transferred to a different unit, and I heard she does not bully anymore. Not sure if it’s because of the law, but it has had an impact on the hospital since the announcements regarding the bullying keep being posted.”*

(Participant 1)

*“I worked at a unit where I spent a lot of time interacting with physicians. Some yelled at and insulted me for my clumsiness; it was all tolerated at that time, but not now. I haven’t heard of this kind of situation at least for a couple of years.”*

(Participant 4)

*“I’m seeing some smiling faces from the newly graduated nurses compared to the past. There were many cases where the new staff were harshly scolded for making mistakes during the handover, but I don’t see that much these days.”*

(Participant 9)

*“I had a terrible experience at my previous workplace, where my senior nurse made me kneel in front of the patient and the family for making a medication error. Instead of having time to learn from my mistake, I was so terrified as a new graduate nurse that I just kneeled and apologized to the patient. I haven’t heard of this kind of cruelty these days.”*

(Participant 8)

#### 3.2.2. Category 2: Need for Awareness of the Law Reform

Category 2 described the participants’ experiences, feelings, and opinions while being on duty after the enactment of the workplace anti-bullying law. It was noticeable that the expressions in this category had negative connotations. The participants expressed regrets and disappointment, and they eased their frustration by sharing their experiences with the researcher (Table 3).

##### Need for Law Reform

The theme “need for law reform” involved the following subthemes: “ambiguous criteria”, “need for awareness about the law”, “lack of knowledge about the application of process”, and “need for strengthening the regulations”.

On gaining some experience since the law has been enacted, the nurses shared the opinion that the law needed to be improved. First, since the term “harassment” involves various subjective factors, one of the issues seemed to be the lack of criteria to identify bullying. Second, some participants also said that there needed to be improvements regarding the awareness of the law after its recent introduction. That is, the nurses thought that workplace bullying could be resolved through improvements in awareness about the law, rather than by taking legal action.

The nurses also reported that although they and their peers had received education regarding the workplace anti-bullying law and prevention of harassment, different forms of harassment continued to exist in practice. They expressed that it was difficult to decide whether they should report the incident, as it was difficult to judge whether the harassment they witnessed or experienced corresponded to the definition provided in the law.

Furthermore, some participants suggested that it was necessary to reinforce the regulations and punishment. That is, if the harassment issue could not be resolved in an autonomous way, then the issue might be resolved by implementing coercive administration. For these reasons, the nurses addressed the need for law reform and discussed the drawbacks and possible improvements regarding the law.

*“It’s hard to report the case since I have no knowledge of whom to contact and what the procedure is…, the law still leaves much to be desired to apply it in reality.”*

(Participant 1)

*“I wish to know the sample scenario in the reporting guidelines. The severity of workplace bullying in nursing is normally less than the actual cases we learn about in education.”*

(Participant 3)

*“I hope there is a stronger punishment for workplace bullying. I believe people will quit bullying as they become afraid of the punishment. It’s similar to the case of the ‘Min-sik Law’, which imposes heavy punishment for school zone traffic offenses. People are now aware of the law and have become more cautious.”*

(Participant 5)

*“I’m not sure against which type of bullying legal action can be taken. Sometimes I’m afraid I might look petty, so I become hesitant to report even the smallest things. It’s especially hard to report as a witness, not as the victim, because I wasn’t sure intervening was the right thing to do at that moment.”*

(Participant 6)

##### Disappointment Caused by Frustrated Expectations

In the theme “disappointment caused by frustrated expectations”, the following three subthemes were identified: “distrust of punishment”, “lack of legal influence”, and “psychological burden of reporting the bully”.

Although the nurses had high expectations about the eradication of bullying in the workplace under the new law, the reality did not meet their expectations; the influence of the law was rather limited. The nurses expected training on the bullying prohibition and prevention law to bring about some positive changes; however, they were hesitant to report the bully, because they feared potential retaliatory repercussions or did not know the appropriate procedure for reporting bullying. The training, which included “one hour training per year”, was not sufficient for the nurses to fully understand and learn the law.

The nurses experienced difficulties in applying the acquired knowledge—provided by a private company that visited the nurses’ workplaces—in real situations. The expectations had gradually lowered, as the participants did not know which department a case of bullying in their workplace should be reported to, and they were not certain that the case would be resolved after reporting.

*“One of the nurses witnessed a bullying scene and reported it to the unit manager. Rather than ensuring confidentiality, the manager talked with all senior nurses one by one about the matter. Later, I have sensed some changes in attitude toward that bullied nurse and others trying to keep a distance from her. The law doesn’t seem to truly eradicate the bullying.”*

(Participant 11)

*“Anything related to the law puts pressure on us. When I think of the law, it reminds me of the whole process of hiring a lawyer, gathering some evidence, and battling for a long period of time. Since every workday is an exhausting battle, I want to relax after work, and not think about anything. So, I’m just living my regular life, hoping that nothing would happen and that I would not have to confront the bully during the handover time. This is why the nurses feel the burden of reporting.”*

(Participant 8)

*“Despite the introduction of the law, the changes are yet to come. My preceptor and I both experienced hazing, but the experience of bullying always takes new forms with newly graduated nurses. While there was some physical bullying, like slapping, in the past, today, the new staff are bullied with verbal insults. Maybe it’s too early to expect some positive changes, I don’t know.”*

(Participant 7)

*“My colleague was once absent from work without any notice because of having been bullied and later told the unit manager about the bullying episode. However, all nurses in that unit knew, and bad rumors were spreading across the hospital. From what I heard, some statements were critical, some were exaggerated, and some rumors were distorted. Ultimately, my colleague was transferred to another unit and had a hard time for several months. Looking at this case, the way workplace bullying is handled is very poor.”*

(Participant 6)

#### 3.2.3. Category 3: Impracticalities of the Law That Caused Chaos

Participants shared their opinions about and experiences in this category more than regarding other categories. They shared all their complaints that had been accumulating. While the participants previously expressed their disappointment about the different perceptions of the law, in this category they talked about their experiences in the workplace in the aftermath of the law (Table 4).

##### Contradictions

The subthemes in this category were “meaningless law and chaos”, “ironic situations caused by the new changes”, and “degenerated culture”. The enactment of the workplace anti-bullying law brought a major disappointment for the nurses. They said that it was more disappointing because their managers, who should serve as role models, could take retaliatory action. In addition, there were situations when experienced nurses had to sacrifice work time for junior nurses, including new graduates. This turned into an ironic situation where the sacrifice of senior nurses was taken for granted.

The essence of the workplace anti-bullying law is to prevent bullying and, if this fails, to protect employees from bullying. Ironically, the law appeared to cause nurses to experience a new form of bullying, rather than protect them from it. The nurses reported that these side effects led them to question who the beneficiaries of the law should be.

*“I think the workplace anti-bullying law has brought about another type of bullying. Once the workplace becomes considerate to the new graduates, I feel the hazing now targets junior nurses who have just begun to get used to the work. Also, I normally get off late due to this law. Even if the nurse on the previous shift has not completed the work properly, I can’t order her to stay until the work is done because it might sound like I’m bullying. So, the remaining work is all mine. Plus, I have my work, which means I would have to work overtime. From my standpoint, it’s hard to report this as workplace bullying.”*

(Participant 3)

*“Since reporting for work early and leaving late was an issue, it is critical to ensure the new graduates’ working time. The problem is that there is no progress in the work. We must report early to the unit because one of our duties is to check the number and quality of the equipment before starting the actual work. The new graduates are not able to finish their work on time without checking the equipment in advance. I’ve had new staff who have been working for more than several months and still do not know where all the equipment and kits are located. With loads of unfinished work, the new graduates call it a day, and the remaining nurses are burdened with the work. Maybe reporting on time might be acceptable but leaving on time without fulfilling your responsibilities doesn’t make any sense.”*

(Participant 7)

*“While on duty, my colleagues and I were approached by my unit manager with a handful of papers; the manager asked us to write down the appropriate ways to communicate and behave based on the bullying case that my colleagues and I were involved in. Some nurses were so preoccupied with work that they did not even have time to sit down or eat. They had to pause their work to write it down, which made them leave late. I thought to myself, ‘Was it really necessary to make us do that during our busy time?’, and ‘Why would I be the person to describe appropriate words and behaviors when I was the victim of the case?’”*

(Participant 1)

##### Purposeless Behaviors

Some nurses showed purposeless behaviors such as “bystander attitude” and “resisting”. It was observed that the experienced nurses tried to read the thoughts of new nurses and allowed them to leave on time even if they had not finished the work. These adverse effects made senior nurses experience difficulties and caused them stress due to the new work burden.

*“The senior nurses often say, “You may leave, and I’ll do the rest”. That being said, I can’t tell the new graduate nurses to stay and finish the work. My workload has doubled, and I sometimes want to cry.”*

(Participant 12)

*“I’ve once witnessed a senior nurse yelling at a new graduate nurse, which was such a surprise that I even dropped something. I know the senior did that only for education purposes, but the new nurse cried and eventually quit the job. Although it was the new graduate’s fault, I wasn’t treated that way when I made the same mistake. I think the senior nurse would not recognize that she is the perpetrator of bullying.”*

(Participant 10)

*“The work atmosphere is different when the unit is filled with the new staff. I’ve noticed some nurses who do not pay attention to the new graduates until they asked for help first. Those nurses are just not trying to look or sound bossy, so they purposely don’t explain something or help the new staff. It’s sad to see the senior nurses quietly come to us [junior nurses] to help them instead.”*

(Participant 2)

## 4. Discussion

The purpose of this study was to understand and explain the meaning of nurses’ experiences by in-depth analysis of nurses’ experiences in the process of implementing anti-bullying laws at work. To this end, 12 nurses currently working in hospitals were selected and interviewed, and the results were derived through Van Kaam’s phenomenological analysis based on the contents. The results of this study were classified into 14 sub-themes, 5 themes, and 3 categories, which were described as “positive effects of the law”, “need for awareness of the law reform”, and “the impracticalities of the law that caused chaos”.

In this study, it was found that the nursing culture regarding workplace harassment has changed and experienced a positive effect after the enactment of the workplace anti-bullying law. It was a positive outcome to be able to experience changes with the enactment of the law in the patterns of nursing bullying and harassment. The positive effect of this law shown in the results of this study was in line with the result of a survey that found that the bullying culture has decreased by 39.2% since the enactment of the law [32].

Positive effects after the application of anti-bullying laws included reduced burnout and stress for new nurses, less bullying of each other, maintaining good communications and a cooperative attitude while working, and helping each other as a whole. These changes seemed to reduce problems such as turnover and physical distress resulting from negative consequences by enabling colleagues to understand each other and work in a bright atmosphere. In addition, factors affecting these outcomes included the manager’s interest and efforts under the prohibition law, and changes in individual behavior and perceptions of nurses could be seen as an important factor of the impact. Therefore, it was explained that the implementation of the anti-bullying law in the workplace was a part where people could directly experience positive changes in the field of nursing.

According to a global nurse study, nurses are at high risk of exposure to workplace bullying in the nursing profession, and workplace bullying harms the nursing environment and work in the same context as this study [4,33,34,35]. In addition, as also could be seen from the results of this study, it was reported that the improvement of the working environment for workplace bullying by nurses had a positive effect on the safety of patients and nurses [4,33,34]. Therefore, it can be seen that the change in nursing group culture and improvement of the working environment under the Workplace Bullying Prevention Act can lead to more positive outcomes, including patient safety.

On the other hand, there was a limit to the application of the workplace harassment ban to the nursing organizational culture. Nurses expressed the opinion that the application of legal standards was still ambiguous with regards to nursing, and that this creates a rather confusing situation about the roles and tasks of individuals.

Oh [35] reported that the workplace harassment ban requires efforts by the users, managers, and workers in a workplace and that since the communication process is important, continuous efforts are needed. Therefore, nurses, managers, and hospital unions should all be attentive to the law and cooperate to become more effective and active in the field.

In addition, nurses mentioned the necessity of education regarding the law. This shows that the provisions to prevent workplace harassment are unclear and that the specified laws of the MOEL that can be applied in the field are insufficient in the clinical field. Since improving recognition can enhance behavior, it is necessary to raise the awareness of workplace bullying in society as a whole [36], which will lead to increased interest in the prohibition of workplace bullying.

The results of this study explained that the influence of the law should be further improved, and legal regulations should be strengthened so that nurses can eradicate workplace harassment. This supports the findings of follow-up surveys by ordinary workers that more stringent penalties are needed to eradicate workplace harassment [37]. In addition, Kim’s [38] report confirmed that the success rate of legislative application was low, there was no pressure on the group, and there was a lack of influence when there was distrust of punishment, similar to the results of this study. Therefore, to increase the reporting rate and prepare institutional strategies and education, it is necessary to increase access from the perspective of users. This would ensure better enforcement of the law in the clinical setting.

Further, this study found that while there are factors that can have a positive effect on the prevention of bullying in the workplace, there are still factors that can lead to negative consequences that confusion. For example, a nurse with experience implementing anti-bullying laws at work explained that bystander attitudes, indifferent behaviors, and limited communication problems always arise to reduce behaviors that could be identified as perpetrators.

In this way, the results of the application of harassment laws not only show disappointment, negative, contradictory, and paradoxical phenomena in the nursing environment, but also have the potential to change the nursing practice culture into a negative situation due to confusion in the nursing environment. However, while the penalties and legal regulations for nurse harassment are important, problems with the structurally existing power in the hospital culture, and the prejudice and behavior of patients and their families, are also important factors of nurse harassment. Therefore, above all, there is a need to first change the overall social perception structure that recognizes the professionalism and independence of nurses in the medical environment.

The limitation of this study was that the time when the anti-bullying law in the workplace was applied and the time when the research was conducted were too close to derive various and extensive empirical contents. In addition, there was a limit to explaining the results of this study, as the contents are commonly experienced in all hospital environments. Therefore, based on sufficient experience in the future, repetitive studies are needed that can interpret and explain specific situations in detail, and studies that can understand deeper and broader experiences by classifying participants in detail according to each characteristic are necessary.

## 5. Conclusions

This study explored clinical experiences based on the in-depth statements of nurses about what occurred in the workplace after the enactment of the anti-bullying law. Based on subjects’ statements, the phenomenological analysis method yielded results in three categories: “positive effect of the law”, “need for awareness of the law reform”, and “impracticalities of the law that caused chaos”.

After the enactment of the workplace harassment ban, the nursing organizational culture with regards to bullying in the workplace seemed to have changed in nursing practice, and showed some positive effects. The experience of changing the environment and acts of bullying in nursing based on the regulations of the law can be the beginning of the process of eradicating bullying in the workplace. This can result in positive effects such as an improvement in the quality of nursing job performance by reducing the turnover rate and improving the organizational culture.

On the other hand, there were also statements that the bullying prohibition in the workplace was not effective, as there were “impracticalities of the law that caused chaos” in the field. This could be due to the natural trial-and-error process of a law that has been introduced recently; although nurses were educated about the law, it seems that they felt difficulties in applying and practicing the law. Based on this situation, however, if appropriate education tailored to the characteristics of the nursing profession is strengthened and the need for recognition improvement is emphasized, we can still find positive signs that the problem of workplace bullying in the field of nursing can be easily resolved.

The results of this study showed the need for additional measures to prevent the workplace bullying of nurses, as well as to respond more actively when bullying occurs. This study highlighted the importance of providing education on legislation banning workplace harassment in an easier way for nurses to understand the process of applying it in their field of practice and the need to supplement this legislation. These results could be used as basic data for the development of an effective education program about the prevention and prohibition of the harassment of nurses in the workplace that could reduce the difference between the provisions of the law and actual experiences in the field.

In particular, in recent years there has been controversy over the ethical awareness of bullying worldwide and the reasons for monitoring and managing bullying and harassment in the workplace in nursing settings, and programs and methods to mitigate bullying through legal mechanisms continue to be developed.

However, since anti-bullying laws have been implemented in the field of real nursing, papers analyzing the results worldwide are scarce. Therefore, since this study has already described nurses’ experiences after the application of the law and explained problems or alternatives based on this, it is meaningful in providing a theoretical basis for applying the results and suggesting a practical direction for enforcing the law.

## Figures and Tables

**Table 1:** Overview of the findings.

Categories	Theme (Number of Significant Statements)	Subtheme (Number of Significant Statements)
Positive effect of the law	Changes in organizational culture in nursing (14)	Changing nursing environment (12)
		Experimental change (2)
Need for awareness of the law reform	Need for law reform (17)	Ambiguous criteria (5)
		Need for awareness about the law (3)
		Lack of knowledge about the application of process (4)
		Need for strengthening the regulations (5)
	Disappointment caused by frustrated expectations (11)	Distrust of punishment (4)
		Lack of legal influence (4)
		Psychological burden of reporting the bully (3)
Impracticalities of the law that caused chaos	Contradictions (39)	Meaningless law and chaos (14)
		Ironic situations caused by the new changes (12)
		Degenerated culture (13)
	Purposeless behaviors (9)	Bystander attitude (5)
		Unresisting behavior (4)

**Table 2:** Positive effect of the law.

Significant Statement	Subtheme	Theme	Categories
“Newly graduated nurses these days are more active and less discouraged”.	Changing nursing environment (12)	Changes in organizational culture in nursing (14)	Positive effect of the law (4)
“What has been considered overlooked and tolerated in the past is not the case today”.			
“The hazing culture (“taeum”) is not completely eradicated, but it is somewhat starting to improve”.			
“I heard that the nurse who bullied has been transferred to another unit and does not repeat the action there”.			
“I would say there has been less vertical conflict with doctors compared to the past”.			
“Nowadays, nurses are more supportive of each other when assigned with challenging tasks”.			
“There has been more care for and support given to newly graduated nurses”.			
“I think the nurses are more satisfied with their job and laugh more than they used to”.			
“It is rare to see nurses being scolded in a public space nowadays”.			
“Nowadays, it is rare to see the hazing culture like in the past, which was totally against common sense”.			
“Whenever there was an announcement regarding prohibiting workplace bullying along with its prevention by the hospital, our unit manager always tried to remind us about this matter”.			
“Newly graduated nurses are not absolutely subject to being bullied anymore”.			
“The law somehow affects nursing culture”.	Experimental change (2)		
“Although I’m not sure whether it’s because of the workplace anti-bullying law or simply just that time has passed, but the atmosphere in the hospital has become tranquil”.			

**Table 3:** Need for awareness of the law reform.

Significant Statement	Subtheme	Theme	Categories
“Although the severity or the type of bullying might be subjective, I believe there has to be some sort of criteria for reporting or giving punishment”.	Ambiguous criteria (5)	Need for law reform (17)	Need for awareness of the law reform (28)
“A new type of workplace bullying is being created, since there are no clear criteria”.			
“I wish to know the sample scenario in the reporting guidelines”.			
“What is the criterion for punishment with regards to the workplace anti-bullying law?”			
“I was provided the education, but it seems challenging for the law to be applied to the hazing culture (taeum)”.			
“If the awareness about the law is raised, the bullying problem will be solved”.	Need for awareness about the law (3)		
“Despite the law enforcement, lack of awareness remains a problem”.			
“I’m concerned that some of the behaviors that used to be okay might be a problem now”.			
“I believe people rarely know about the workplace anti-bullying law”.	Lack of knowledge about the application of process (4)		
“It’s hard to report the case since I have no knowledge of whom to contact and what the procedure is”.			
“Would certain behaviors without malicious intent due to high intensity of work be considered as bullying as well?”			
“I’m not sure about the criterion in which the workplace anti-bullying law applies to everyone regardless of their work experience”.			
“I hope to see more strengthened regulation as well as punishment”.	Need for strengthening the regulations (5)		
“The severity of workplace bullying in nursing is normally less than the actual cases we learn about in education”.			
“Once you report the incident, I feel that progress would be slow in dealing with it”.			
“I think it’s hard to call it a substantial law”.			
“The law still leaves much to be desired to apply it in reality”.			
“I know there has been a bullying case reported, but I have never heard of any actions taken in response to the incident”.	Distrust of punishment (4)	Disappointment caused by frustrated expectations (11)	
“Regardless of the law enforcement, I’m still afraid the hazing culture might continue to exist or be passed on”.			
“I feel that it would take a great deal of time for the law to be stabilized”.			
“I have not yet seen the case of nurses regarding the workplace anti-bullying law”.			
“My colleague once reported to her unit manager that she was being bullied, but the actions taken weren’t really helpful”.	Lack of legal influence (4)		
“I just hope everyone gives up on fighting with each other and squashes such issues as it only wastes time rather than taking legal action”.			
“No changes have been made despite the existence of the law”.			
“There was a drawback in the law where the bully was reported and eventually left the workplace”.			
“What if the victim does not want the witness to report the bully?”	Psychological burden of reporting the bully (3)		
“The term ‘law’ itself makes one disinclined to report”.			
“With the workload, it creates more pressure to report and deal with the whole situation”.			

**Table 4:** Impracticalities of the law that caused chaos.

Significant Statement	Subtheme	Theme	Categories
“The unit manager still treats me differently despite the enactment of the workplace anti-bullying law”.	Meaningless law and chaos (14)	Contradictions (39)	Impracticalities of the law that caused chaos
“Although working overtime is voluntary, my unit manager reaffirmed that it was necessary”.			
“Many suggested improvements that seemed so obvious”.			
“After reporting for duty, I was given a retaliatory work schedule excluding the days I wanted to work”.			
“The unit manager’s indecisive manner fails to set an example for law enforcement”.			
“The education programs about the law provided by the hospital were not considered”.			
“When workplace harassment occurs, there are more cases where the victim gets transferred to another workplace, rather than the perpetrator”.			
“I learn a lot of nursing tasks from the handover, but removing this process simply because of it being a potential cause of hazing is not right”.			
“‘Reducing workload’ to prevent workplace bullying delays learning the tasks”.			
“Part of the improvements were nothing but window dressing”.			
“Even though the unit manager and the senior nurses try their best to adapt to the improvement, some nurses make no effort”.			
“It’s such a nonsensical idea to ask the victim to write an incident report for the perpetrator as a suggestion”.			
“The hazing culture still exists as an excuse for education”.			
“I’m not sure if doubling the workload was the right solution for the improvement”.			
“From a third-party perspective, I might appear as a perpetrator; although, in reality, I am the victim”.	Ironic situations caused by the new changes (12)		
“In a situation where I need to say something to the junior nurses, I’m not sure whether I am being a bully or being bullied”.			
“People are becoming more cautious about correcting the mistakes made by the nurses”.			
“Apparently you wouldn’t consider workplace bullying and file a report in a situation where my workload has doubled because of the previous staff leaving without finishing their duty”.			
“Being not able to fulfill my basic needs, I got off the unit late, for writing down some improvement plans”.			
“My caring attitude can be seen as excessive monitoring of others”.			
“Newly graduated or junior nurses having trouble getting used to the task might be attributed to the senior nurses”.			
“I thought to myself, ‘Why do I, the bullying victim, have to come up with an improvement plan?’ ”			
“The one who gave us the unmanageable workload is not claiming herself to be the victim and considering us to be the perpetrators”.			
“I’m afraid that I might be the subject of harassment. But if holding back emotions becomes a hospital norm, then the law becomes meaningless”.			
“If reporting workplace bullying doesn’t work out well, all the backlash goes to the victim”.			
“The workplace bullying does not always occur between vertical relations”.			
“I don’t feel comfortable working in a place where I have to be cautious of every single behavior to prevent workplace harassment”.	Degenerated culture (13)		
“Nurses nowadays, especially the newly graduated ones, think getting off on time is a must without exceptions”.			
“The atmosphere changes when the unit is filled with newly graduated nurses on the same shift”.			
“I felt betrayed”.			
“The drawback of bullying at the workplace is a lack of communication among the nurses”.			
“It becomes a problem even if we cannot get off work simply because we have remaining tasks to be done”.			
“Fearing being accused of being the perpetrator, I was not able to fully communicate with other staff and got off work late”.			
“The nurses don’t have an appreciative mind even if others notice the incompetency and help you out”.			
“Some would call it a day without completing the given task”.			
“I’m better off working by myself than helping others”.			
“If the nurse from the previous duty is a new graduate, the workload doubles, but it’s something I have to deal with”.			
“It’s kind of frustrating to see nurses obsess over getting off on time, not having a sense of responsibility”.			
“The presence of inexperienced staff causes the other workers’ workload to double”.			
“There isn’t a person who gives advice to either the bully or the bullied”.	Bystander attitude (5)	Purposeless behaviors (9)	
“The nursing work environment still needs to be improved”.			
“There was a case when the problem got naturally solved by nurses moving to another department or workplace without any action taken”.			
“Despite the law enactment, some nurses would still yell at others for training purposes”.			
“It would be a problem if someone left the workplace due to our bullying, so it is best not to intervene in any situation”.			
“The sacrifice and solicitude to eradicate workplace bullying should not be considered as obvious, rather out of gratitude”.	Unresisting behavior (4)		
“I become more cautious even when lending a hand. I prefer not to work with inexperienced staff”.			
“It’s a bit frustrating that certain ideas that used to be questioned without a doubt can now be a potential cause of bullying”.			
“I believe there are no bullies bullying keeping in mind that they will be reported someday”.			

## Data Availability

The data are not publicly available due to ethical reason.
